# 4‑Fold Protonation of Tetracyanometalates in Superacids: Hydrogen and π‑Hole Bonding in the Solid State

**DOI:** 10.1021/acs.inorgchem.5c05224

**Published:** 2025-12-29

**Authors:** Tim-Niclas Streit, Malte Sellin, Susanne M. Rupf, Rosa M. Gomila, Antonio Frontera, Moritz Malischewski

**Affiliations:** † Institut für Chemie und Biochemie, 9166 Freie Universität Berlin, Fabeckstraße 34−36, 14195 Berlin, Germany; ‡ Department of Chemistry, 27209 University of Basel, St. Johanns-Ring 19, 4056 Basel, Switzerland; § Departament de Quimica, 16745 Universitat de les Illes Balears, Crta. de Valldemossa km 7.5, 07122 Palma de Mallorca (Baleares), Spain

## Abstract

Reaction of the group 10 tetracyanometalates K_2_[M^II^(CN)_4_] (M = Ni, Pd, Pt) and tetracyanoaurate K­[Au^III^(CN)_4_] with an excess of the superacid HF/SbF_5_ results in the formation and structural characterization of homoleptic hydrogen isocyanide complexes [M^II^(CNH)_4_]­[SbF_6_]_2_ (M = Ni, Pd, Pt) and [Au^III^(CNH)_4_]­[SbF_6_]_3_·2HF, respectively. The intermolecular interactions in the solid state are dominated by strong H···F bonded networks as well as weak contacts between the fluorine atoms and C≡N groups, which are more pronounced for the more electrophilic trication. Additionally, M···F contacts below the sum of van der Waals radii for all compounds are observed, which can be regarded as regium bonding. Furthermore, density functional theory (DFT) calculations were performed to provide an in-depth energetic and electronic characterization of the observed M···F interactions. Molecular electrostatic potential (MEP) surfaces confirm the existence of a π-hole (electrophilic region) over the metal centers, a notable transformation for these typically nucleophilic square-planar complexes of Ni^II^, Pd^II^, and Pt^II^. Quantum theory of atoms in molecules (QTAIM) analysis confirms the noncovalent, closed-shell nature of the M···F contacts. Additionally, natural bond orbital (NBO) analysis quantifies the donor–acceptor character of these regium/π-hole interactions.

## Graphical abstract

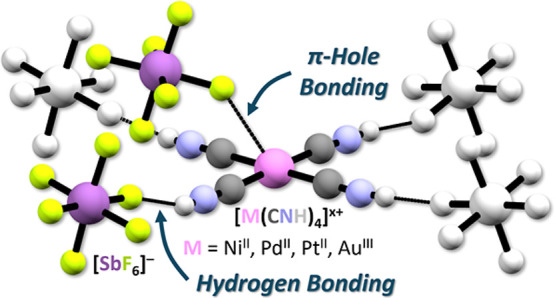

## Introduction

Due to their diverse, but rigid topologies, cyanometalates [M­(CN)_*x*_]^*z*−^ are ubiquitous building blocks in coordination chemistry,^1^ as they can serve as multitopic nucleophiles with distinctive geometries. In combination with Lewis-acidic metal cations, they form network structures M–CN–M which are of interest for batteries,^2,3^ as well as porous^4^ and magnetic materials.^5,6^ Recently, cyanometalates have been increasingly used as Lewis-basic building blocks for noncovalent interactions, e.g. in combination with halogen-bond,^7–10^ chalcogen^11,12^ and hydrogen bond donors.^13–18^ If taken to the extreme, a very strong hydrogen bond could lead to the protonation of the cyano ligand, leading to the formation of hydrogen isocyanide complexes M-CNH.^19,20^ However, the protonation of cyanometalates is relatively little explored due to safety concerns related to the potential release of HCN in case of the decomposition of the complexes. A few examples of homoleptic hydrogen isocyanide complexes have been reported. Especially noteworthy is the Fe­(II) complex [Fe­(CNH·O­(H)­Et)_6_]­[Cl]_2_ in which the [Fe­(CNH)_6_]^2+^ moiety is stabilized by strong hydrogen bonds to six adjacent ethanol solvent molecules.^21^ Recently, we reported the isolation and structural characterization of [M­(CNH)_8_]­[SbF_6_]_4_·2 HF (M = Mo, W) by the reaction of K_4_[M­(CN)_8_] with the superacidic combination of anhydrous hydrogen fluoride (HF) and antimony pentafluoride (SbF_5_).^17^ Despite the pronounced electrophilicity of the metal centers in such 6-fold and 8-fold coordinated complexes, the electrophilic metal center is too shielded to form contacts with anions.

In the past years, the analysis of noncovalent interactions between electrophilic transition metal centers and Lewis-basic donor groups has received increased attention.^22,23^ In this context, the term regium bonding has been introduced for group 11 metals;^24–27^ however, the term was later also extended to group 10 metals.^23^ For the latter, the differences between the lighter and the heavier elements are of particular interest. For example, a higher electrophilicity of the nickel­(II) compound in comparison to Pd­(II) and Pt­(II) would be expected.^28,29^ Concerning metal centers with a d^8^ electron configuration, significantly fewer copper and silver compounds are known in oxidation state +III than for gold. For Au­(III), the formation of contacts between the π-hole on the metal center and electron-rich donor sites has already been described as regium bonding.^30–34^ These interactions turned out to be highly relevant for metal-protein interactions.^35–38^

As we had already demonstrated the complete protonation of all ligands in cyanometalates by the superacid HF/SbF_5_,^17^ we wondered whether the complexes [M^II^(CNH)_4_]^2+^ (M = Ni, Pd, Pt) and [Au^III^(CNH)_4_]^3+^ could be synthesized by a similar approach and whether the strongly positively charged (and sterically accessible) metal centers would form contacts to the weakly coordinating [SbF_6_]^−^ anions. It is noteworthy that square-planar d^8^ complexes of Ni^II^, Pd^II^, and especially Pt^II^ are generally considered to be nucleophilic due to the high-lying lone pair in the d_z^2^_ orbital. This inherent nucleophilicity facilitates their well-documented participation as H-bond and halogen-bond acceptors. The 4-fold protonation strategy used here is expected to dramatically reduce the electron density at the metal center, thus inducing a “supramolecular Umpolung” where the core of the system is switched from an electron donor to a potent electron acceptor. This fundamental change allows the electrophilic metal center to engage in noncovalent interactions, specifically the π-hole regium bonds, with the weakly coordinating anion. To confirm this electronic transformation and provide a rigorous characterization of the M···F contacts, we have utilized density functional theory (DFT) calculations.

## Results and Discussion

Reacting the group 10 cyanometalates K_2_[Ni­(CN)_4_], K_2_[Pd­(CN)_4_], K_2_[Pt­(CN)_4_], and K­[Au­(CN)_4_] with the same reaction conditions resulted in the 4-fold protonation of the tetracyanometalates (Scheme 1).

**Scheme 1:**
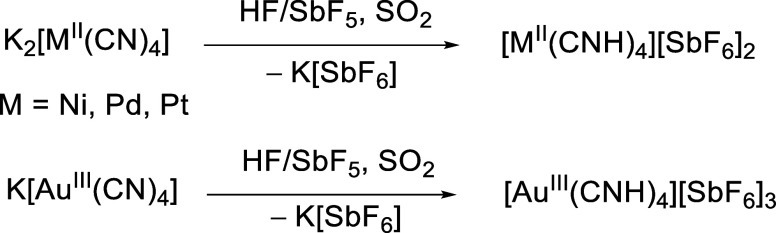
Fourfold Protonation of Tetracyanometalates in the Superacid HF/SbF_5_

Similar to their highly charged early transition metal analogues, the addition of anhydrous SO_2_ was required to bring the di- and tricationic charged hydrogen isocyanide complexes into solution at room temperature. After slowly cooling the reaction mixture to −70 °C, high-quality, colorless crystals could be obtained for crystallographic investigations. [Ni­(CNH)_4_]­[SbF_6_]_2_ and [Pd­(CNH)_4_]­[SbF_6_]_2_ crystallize in the monoclinic space group *C*2/*m*. For [Pt­(CNH)_4_]­[SbF_6_]_2_, the refinement in the monoclinic space group *I2/m* resulted in slightly better crystallographic data. [Au­(CNH)_4_]­[SbF_6_]_3_·2HF crystallizes in the orthorhombic space group *P* *nma.* It is a rare example of an Au­(III) complex featuring only neutral ligands, as shown in seminal works by Dutton.^39–41^ In all cases, refinement with the connectivity M–C≡N–H yielded better crystallographic values than for M–N≡C–H. Exceptional data quality enabled the location of all hydrogen atoms via difference electron density mapping. Interestingly, the M–C≡N–H bond angles vary slightly from the ideal 180°, ranging from 172–176° across all structures. A set of linearly directed strong H···F contacts with a length between 1.76 (12)–1.89(4) Å was observed for all dicationic complexes. For all group 10 structures C≡**N**–H**···F** distances are in the range of 2.618(9)–2.652(2) Å, respectively (Figure 1).

**Figure 1:**
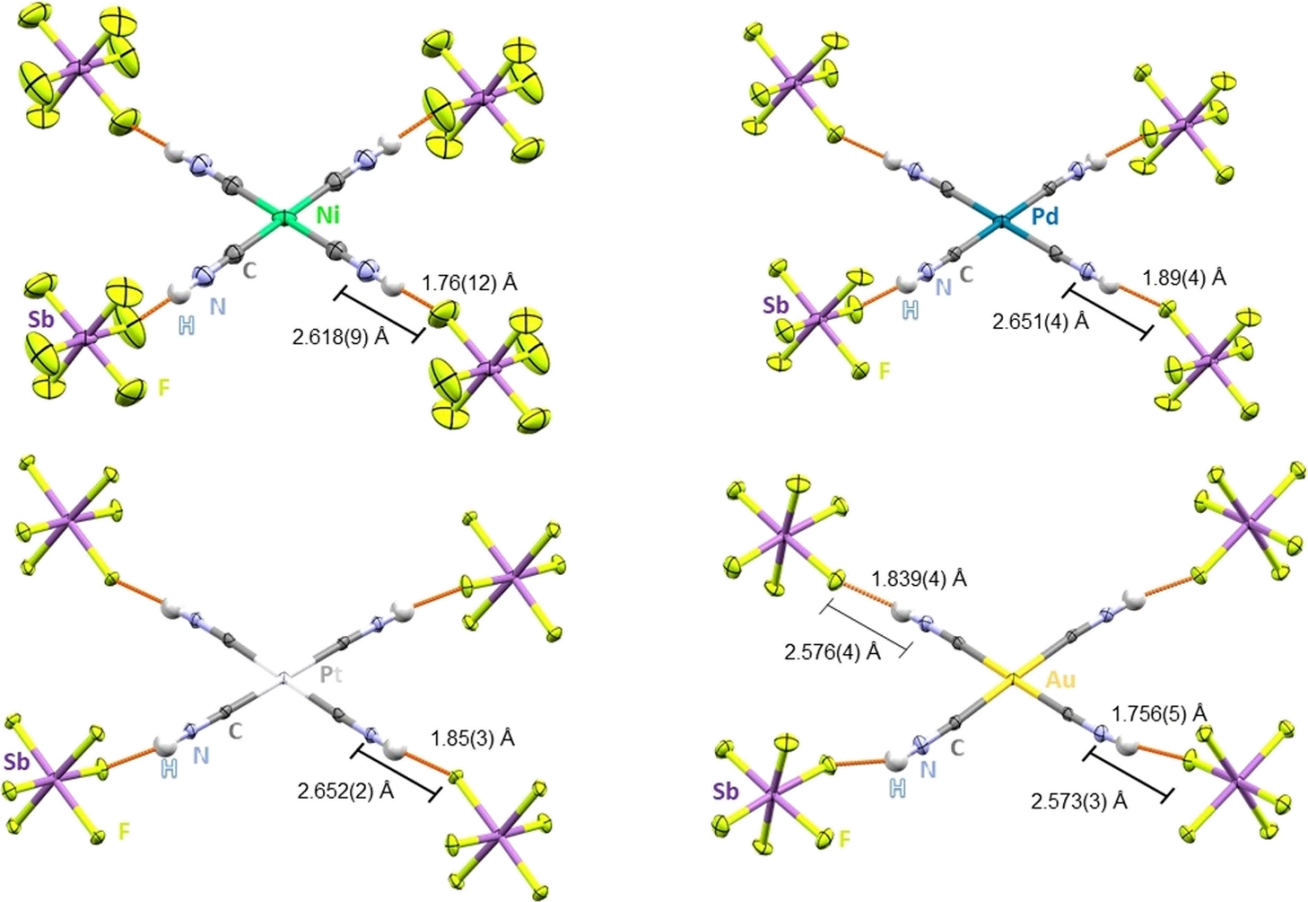
Molecular structures of the hydrogen isocyanide complexes in solid-state determined by single-crystal X-ray diffraction, highlighting the strongest hydrogen bonds (in orange) to the [SbF_6_]^−^ counterions. [Ni­(CNH)_4_]­[SbF_6_]_2_ (top left), [Pd­(CNH)_4_]­[SbF_6_]_2_ (top right), [Pt­(CNH)_4_]­[SbF_6_]_2_ (bottom left) and [Au­(CNH)_4_]­[SbF_6_]_3_·2HF (bottom right). Ellipsoids drawn at 50% probability.

In the case of [Au­(CNH)_4_]­[SbF_6_]_3_·2 HF, the N–H···F angles deviate significantly from 180° and range between 156–168°, and H···F hydrogen bonds of 1.749(6) and 1.887(3) Å were found. C≡**N**–H**···F** distances are shortened compared to the dicationic complexes to 2.573(3) and 2.576(4) Å and are well in agreement with other protonated cyanometalates in the literature.^17^

Comparison of M–C bond lengths in the polycationic complexes [M­(CNH)_4_]^2+^ (M = Ni, Pd, Pt) and [Au­(CNH)_4_]^3+^ reveals only minimal changes in comparison to the unprotonated forms (Table 1). Nonetheless, the 4-fold protonation noticeably influences C≡N bond lengths in these cationic complexes, resembling values close to protonated nitriles. A shortening of the C≡N bond is notable and lies in the range of several picometers. This is also reflected by the IR spectra and Raman spectra of [M­(CNH)_4_]^2+^ (M = Ni, Pd, Pt) and [Au­(CNH)_4_]^3+^, which show an increase of ≈50 cm^–1^ for the C≡N bond, which is in accordance with other homoleptic metal isocyanide complexes.^17^ Furthermore, the presence of four isocyanide (CNH) groups results in the observation of broad bands in the IR spectrum at 3100–3300 cm^–1^ for the CN–H stretching vibrations (Table 2) as well as weak CN–H deformation vibrations at 1600 cm^–1^.

**Table 1:** Comparison of the Key Bond Lengths (Å) of the Homoleptic Hydrogen Isocyanide Complex Salts with Their Parent Cyanometalate Salts

distances in Å	[Ni(CNH)_4_][SbF_6_]_2_	Na_2_[Ni(CN)_4_]·3H_2_O^42^	[Pd(CNH)_4_][SbF_6_]_2_	K_2_[Pd(CN)_4_][Bibr ref43]
M–C	1.857(7)	1.851(1)–1.865(1)	1.985(3)	1.980(7)–1.994(6)
C≡N	1.130(9)	1.158(1)–1.168(1)	1.124(4)	1.180(9)
M–F	2.729(2)		3.146(2)	
CNH···F	2.618(9)		2.651(4)	

distances in Å	[Pt(CNH)_4_][SbF_6_]_2_	K_2_[Pt(CN)_4_][Bibr ref43]	[Au(CNH)_4_][SbF_6_]_3_·2HF	K[Au(CN)_4_]·H_2_O^44^
M–C	1.982(2)	1.980(2)	1.990(3) and 1.997(3)	1.96(1)–1.99(1)
C≡N	1.127(3)	1.150(3)	1.113(4) and 1.124(4)	1.12(1)–1.17(1)
M–F	3.284(2)		2.701(3) and 2.968(3)	
CNH···F	2.652(2)		2.573(3) and 2.576(4)	

**Table 2:** Experimental and Literature IR and Raman Data in cm^–1^

		K[Au(CN)_4_][Bibr ref44]	[Au(CNH)_4_][SbF_6_]_3_	K_2_[Pt(CN)_4_][Bibr ref43]	[Pt(CNH)_4_][SbF_6_]_2_
IR	ν(CN–H)		3148		3307
	ν(CN)	2189	2212	2137, 2129	2190
Raman	ν(CN)	2207, 2198		2168, 2146	2221, 2205

		K_2_[Ni(CN)_4_][Bibr ref45]	[Ni(CNH)_4_][SbF_6_]_2_	K_2_[Pd(CN)_4_][Bibr ref43]	[Pd(CNH)_4_][SbF_6_]_2_
IR	ν(CN–H)		3225		3290
	ν(CN)	2120	2181	2159, 2150	2190
Raman	ν(CN)	2142, 2135	2195	2158, 2147	2213, 2199

Additionally, [M­(CNH)_4_]­[SbF_6_]_2_ and [Au­(CNH)_4_]­[SbF_6_]_3_·2HF exhibit significant M···F contacts of 2.729(4) Å for the nickel and between 2.701(3) and 2.968(3) Å for the gold compound (Figure 2), which lie significantly below the sum of Batsanov’s van der Waals radii (∑vdW (Ni–F): 3.45 Å, (Au–F): 3.5 Å).^46,47^ Slightly longer M···F contacts were observed in the crystal structures of [Pd­(CNH)_4_]­[SbF_6_]_2_ and [Pt­(CNH)_4_]­[SbF_6_]_2_ but the M···F distances (Pd: 3.146(2) Å & Pt: 3.284(2) Å) are still below the corresponding vdW radii (∑vdW (Pd–F: 3.55 Å, Pt–F: 3.55 Å).^46,47^

**Figure 2:**
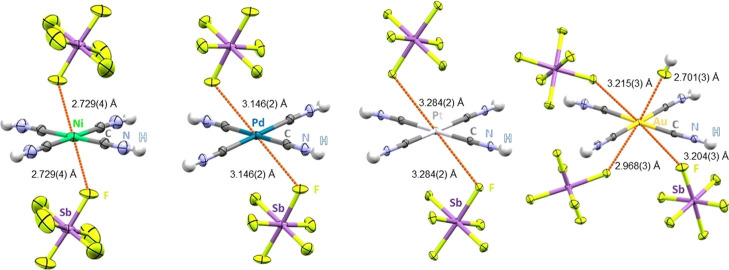
Selected M···F contacts (in orange) and bonding angles in the molecular structures in solid state of [Ni­(CNH)_4_]­[SbF_6_]_2_, [Pd­(CNH)_4_]­[SbF_6_]_2_, [Pt­(CNH)_4_]­[SbF_6_]_2_ and [Au­(CNH)_4_]­[SbF_6_]_3_·2HF. Ellipsoids depicted at 50% probability.

In all structures, there are common geometrical features regarding the M···F contacts (Figure 3, Table 1). The fluorine atoms always lie on the bisector between two CNH ligands (θ = 45°). The angle λ of the M···F contact to the square-planar di- or tricationic [M­(CNH)_4_] moiety is always significantly below 90° and decreases in the order 3d > 4d > 5d. Similarly, the shortest M···F contacts (d) were observed for Ni­(II) in the case of group 10 metals and in the case of Au­(III) due to increased electrostatics.

**Figure 3:**
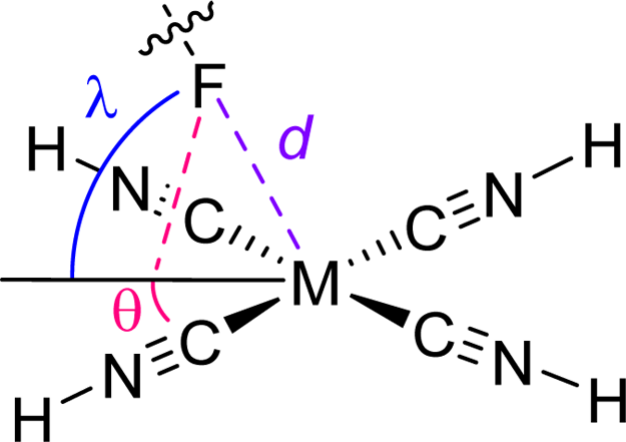
Geometrical parameters describing the M···F contacts.

While the crystal structures unequivocally confirm the 4-fold protonation and reveal the presence of unusually short M···F contacts, a complete understanding of the nature and strength of these regium/π-hole interactions requires a deeper theoretical investigation. To this end, we performed density functional theory (DFT) calculations on the isolated [M­(CNH)_4_]­[SbF_6_]_*n*_ (*n* = 2 or 3) salts. This approach allowed for a quantitative analysis of the interaction energies (*E*_int_), electronic charge redistribution, and a rigorous electronic characterization. Specifically, we used molecular electrostatic potential (MEP) surfaces to visualize and quantify the π-hole on the metal center. Furthermore, the quantum theory of atoms in molecules (QTAIM) was employed to topologically confirm the noncovalent nature of the M···F and H···F bonds, and natural bond orbital (NBO) analysis was used to characterize the donor–acceptor orbital contributions to the M···F contacts.

To characterize the electronic transformation upon protonation, specifically the emergence of π-holes at the metal centers and σ-holes at the hydrogen isocyanide ligands, we computed the MEP surfaces for the salts, [M­(CNH)_4_]­[SbF_6_]_2_ (M = Ni, Pd, Pt) and [Au­(CNH)_4_]­[SbF_6_]_3_, based on the solid-state geometries (Figure 4). The surfaces reveal the distribution of electrophilic and nucleophilic regions, with key values summarized in Figure 4. The di- and tricationic metal complexes are strongly electrophilic across the entire core, which is consistent with the experimental observation of H···F and M···F contacts with the weakly coordinating [SbF_6_]^−^ counterions. The MEP minimum is consistently located on the [SbF_6_]^−^ anions, ranging from −44.9 to −54.2 kcal/mol, confirming their expected role as Lewis basic sites. For the group 10 divalent complexes (M = Ni, Pd, Pt), the overall most positive regions (MEP maximum) are located at the H atoms of the isocyanide ligands, with values ranging from 127.1 to 128.6 kcal/mol. These high, consistent values confirm the strong H-bond donor ability of the protonated ligands. More importantly, a pronounced π-hole is observed around the metal centers. This electrophilic region represents a significant change from the typically nucleophilic nature of d^8^ square-planar complexes. For Ni, the MEP local maximum for the π-hole is located directly at the metal atom (100 kcal/mol), while for Pd and Pt, the π-hole maximum is shifted above the bisector of the C–M–C bond (≈97 kcal/mol, see lower panel in Figure 4). This finding aligns with the experimental structures where the λ values being closest to 90° (Figure 3, Table 3) were observed for Nickel (76°, see Figure 3, Table 3) and clearly surpass the values of the 4d and 5d metals (60.2–55.9°). Furthermore, it explains why θ angles of 45° (corresponding to the bisector of the C–M–C) were observed for all structures. The electrophilicity of the metal center diminishes upon descending the group, with the MEP at the Ni atom (100 kcal/mol) being approximately 20 kcal/mol greater than the MEP at the Pt atom (81.8 kcal/mol). This electronic trend is in perfect agreement with the experimentally observed M···F distances, which increase from Ni (2.729(4) Å) to Pd (3.146(2) Å) and Pt (3.284(2) Å). As aforementioned, the MEP local maximum is not located over the metal atom for the Pd and Pt complexes, but is instead shifted slightly above the bisector of the C–M–C bond (see lower panel of Figure 4). For the experimental structures, the λ angles are 60.2 degrees and 55.9 degrees, respectively, values that are notably larger than the location of the local maximum. This deviation of the λ angle is governed by a subtle balance of electronic and packing effects. The geometry is influenced by several competing contributions to the total energy in addition to the attraction to the π-hole. This includes the residual influence of the filled d_*z* ^2^_ orbital, which is inherent to the d^8^ square-planar configuration, acts as a Lewis base, and is a repulsive element in the axial direction. This repulsion is particularly pronounced for the heavier elements with larger orbitals (Pd and Pt), contributing to the greater angular deviation. Furthermore, other contributions to the total binding energy, such as dispersion forces and the long-range Coulombic repulsion/attraction with the adjacent counterions in the solid state, also influence the final λ values.

**Figure 4:**
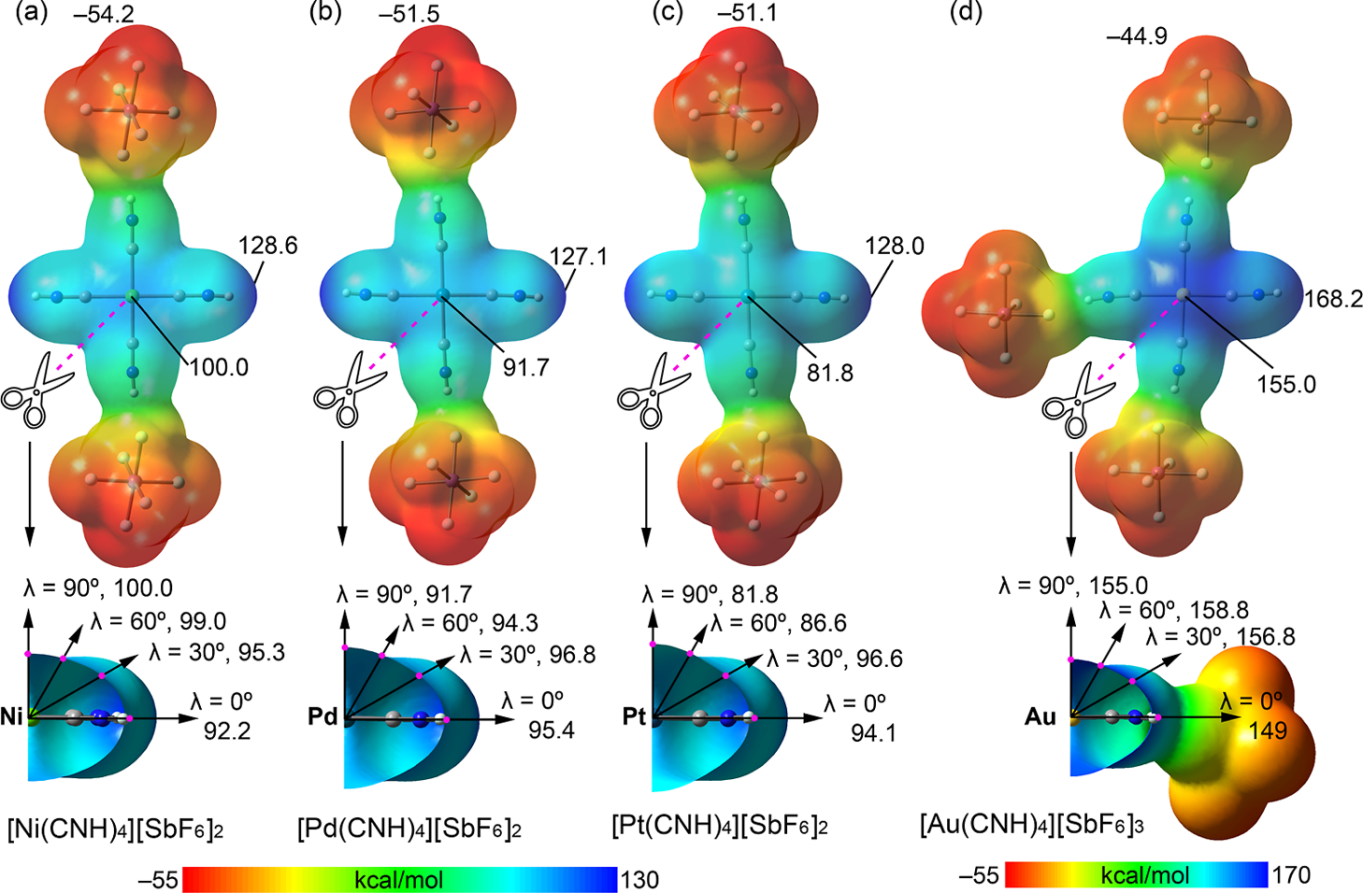
MEP surfaces of the optimized salts [M­(CNH)_4_]­[SbF_6_]_2_ (M = Ni, Pd, Pt) and [Au­(CNH)_4_]­[SbF_6_]_3_. Selected MEP values (in kcal/mol) at the MEP minima (anion, red) and maxima (H atom and π-hole, blue) are shown. The color scale is set in the range −55 to 130 kcal/mol for Ni, Pd, and Pt and −55 to 170 kcal/mol for Au. In the lower panel open surfaces are represented and the MEP values at different values of λ are indicated in kcal/mol.

**Table 3:** Geometrical Parameters Describing the M···F Contacts

	F atom	*d*	θ	λ
[Ni(CNH)_4_][SbF_6_]_2_		2.729(4)	45°	76.3°
[Pd(CNH)_4_][SbF_6_]_2_		3.146(2)	45°	60.2°
[Pt(CNH)_4_][SbF_6_]_2_		3.284(2)	45°	55.9°
[Au(CNH)_4_][SbF_6_]_3_·2HF	F7 (HF)	2.701(3)	45°	64.4°
	F1 (SbF_6_^–^)	2.968(3)	45°	60.6°
	F15 (SbF_6_^–^)	3.204(3)	45°	50.1°
	F8 (SbF_6_^–^)	3.215(3)	45°	46.4°

For the highly charged gold trication, [Au­(CNH)_4_]^3+^, the core exhibits a stronger electrophilicity compared to the group 10 dications. The difference in MEP values between the H atoms (168.2 kcal/mol) and the π-hole region (159 kcal/mol) is small (<10 kcal/mol), confirming that the Au^III^ center is a particularly strong electrophile in this complex, consistent with its shorter Au···F contacts. The analysis along the bisector (Figure 4d, lower panel) shows that the local MEP maximum is located at λ ≈ 60° (≈159 kcal/mol), in line with the experimental values (see Table 3).

To provide a quantitative measure of the strength of the observed noncovalent interactions, we computed the binding energies for the key structural motifs present in the crystal lattices using DFT. The models were based on the experimental X-ray geometries, defining the [M­(CNH)_4_]­[SbF_6_]_2_ (M = Ni, Pd, Pt) and [Au­(CNH)_4_]­[SbF_6_]_3_ moieties as monomers, and calculating the interaction energy of their M···F and H···F contacts within a simplified cluster model (Figure 5). The calculated binding energies are large due to the dominant electrostatic (ion-pair) nature of the interaction. For the divalent cations, the binding energy for the SbF_6_^–^··· [M­(CNH)_4_]­[SbF_6_]_2_ motif weakens subtly on descending the group: −55.5 kcal/mol for Ni, −53.9 kcal/mol for Pd, and −51.4 kcal/mol for Pt. This trend is in excellent agreement with the decreasing electrophilicity observed in the MEP analysis and corroborates the experimental increase in the M···F distances (2.729 Å to 3.284 Å). As anticipated from the Au^III^ trication’s significantly stronger electrophilicity in the MEP analysis (159 kcal/mol), the ion-pair binding energy for the SbF_6_^–^···[Au­(CNH)_4_]­[SbF_6_]_3_ model is substantially larger, calculated at −86.0 kcal/mol. To isolate and estimate the strength of the pure regium bonding interaction, we computed an additional model, HF···[Au­(CNH)_4_]­[SbF_6_]_3_, which features a fluorine atom from an HF molecule at the experimental Au···F distance (2.968 Å) and thus largely removes the strong long-range Coulombic influence of the [SbF_6_]^−^ counteranion. In this neutral interaction model, the binding energy is significantly reduced to −13.2 kcal/mol, providing a good estimate for the intrinsic strength of the Au···F π-hole interaction in the absence of overwhelming ionic contributions.

**Figure 5:**
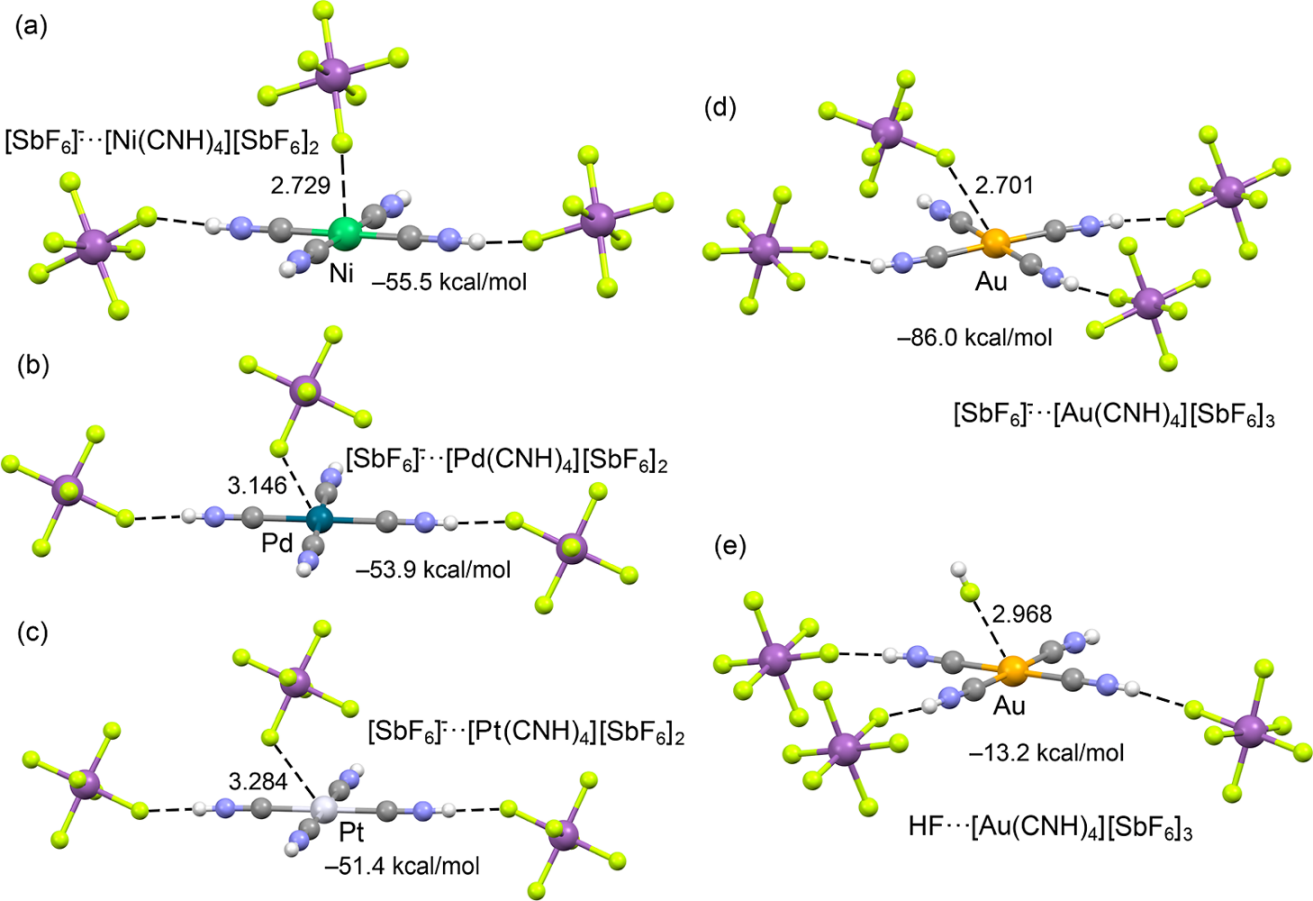
Cluster models and calculated binding energies (kcal/mol) for the M···F interactions based on the geometries derived from single-crystal X-ray diffraction, distances in Å. Models (a–c) show the [SbF_6_]^−^···[M^II^(CNH)_4_]­[SbF_6_]_2_ motif for the group 10 complexes (M^II^ = Ni, Pd, Pt). Models (d,e) show the ion-pair [SbF_6_]^−^···[Au^III^(CNH)_4_]­[SbF_6_]_3_ and the neutral HF·[Au^III^(CNH)_4_]­[SbF_6_]_3_ complex, respectively.

To provide a rigorous topological confirmation of all noncovalent interactions, we performed a quantum theory of atoms in molecules (QTAIM) analysis on the optimized trimeric assemblies (Figure 6). The existence of a bond critical point (BCP), shown as a small red sphere, and a corresponding bond path (orange line) between two atoms is the definitive topological indicator of an interaction. For the group 10 divalent complexes, distinct bonding motifs were observed. The [Ni­(CNH)_4_]­[SbF_6_]_2_ assembly exhibits the most extensive network: each [SbF_6_]^−^ anion is connected to the Ni core via three BCPs and bond paths. One path confirms the direct Ni···F contact, while the other two connect an additional F atom of the anion to two carbon atoms of the hydrogen isocyanide ligands (F···C contacts). In contrast, the [Pd­(CNH)_4_]­[SbF_6_]_2_ and [Pt­(CNH)_4_]­[SbF_6_]_2_ assemblies show a simpler connection, with the anion linked to the metal core via only a single BCP and bond path, unequivocally confirming the M···F contact. For the Au^III^ complex in the ion-pair assembly [SbF_6_]^−^···[Au­(CNH)_4_]­[SbF_6_]_3_, the anion is connected by three BCPs and bond paths: one Au···F contact and two secondary F···N contacts to the hydrogen isocyanide ligands. When the stronger [SbF_6_]^−^ anion is replaced by a neutral HF molecule (model HF···[Au­(CNH)_4_]­[SbF_6_]_3_), a single BCP connects the F atom directly to the Au-atom, confirming the isolated Au···F regium bond. In all cases, the values of the electron density at the BCP, ρ­(*r*), are well below the 0.04 au threshold, which is characteristic of weak, closed-shell, noncovalent bonding. The magnitude of the electron density, ρ­(*r*), is directly correlated with the strength of the interaction and allows for an electronic comparison. Remarkably, for the divalent metals, the ρ­(*r*) value for the M···F contact is greatest for Ni (0.0145 au), followed by Pd (0.0088 au) and Pt (0.0080 au). This trend Ni > Pd ≈ Pt is consistent with the decreasing electrophilicity observed in the MEP analysis and the corresponding increase in experimental M···F distances, confirming that the Ni^II^ center is the most effective π-hole donor among the divalent ions. For the Au^III^ trication, a comparison between the Au···F contacts in the two models is particularly illuminating. The ρ­(*r*) value for the Au···F contact with the neutral HF molecule (0.0209 au) is significantly larger than the ρ­(*r*) value for the Au···F contact with the [SbF_6_]^−^ anion (0.0137 au). This key finding confirms that, in the absence of strong, competing long-range Coulombic effects from the highly charged counteranion, the intrinsic strength of the HF···Au regium bond is greater than the [SbF_6_]^−^···Au interaction, providing a strong electronic argument for the underlying π-hole driving force.

**Figure 6:**
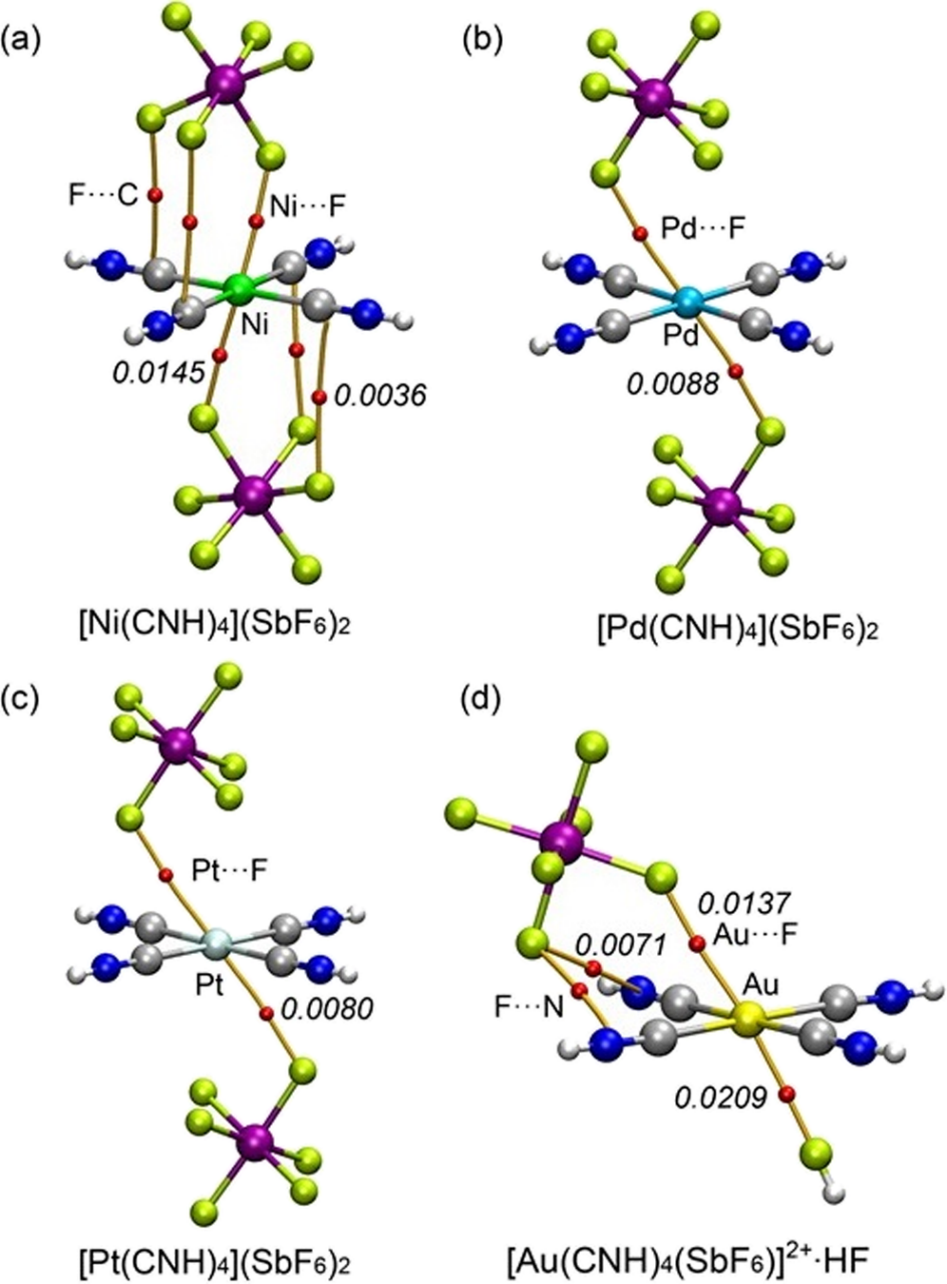
QTAIM analysis of the M···F interactions. The molecular graphs are shown for the trimeric assemblies of (a) [Ni­(CNH)_4_]­[SbF_6_]_2_, (b) [Pd­(CNH)_4_]­[SbF_6_]_2_, (c) [Pt­(CNH)_4_]­[SbF_6_]_2_, and (d) the Au^III^ model [Au­(CNH)_4_(SbF_6_)]^2+^·HF. BCPs are represented by small red spheres, and bond paths are shown as orange lines. The electron density at the BCP, ρ­(*r*) (in a.u.) is indicated in italics.

The natural bond orbital (NBO) analysis was performed to quantify the electronic origin of the M···F regium/π-hole interactions by calculating the charge transfer between the donor and acceptor orbitals. In all studied complexes, the primary orbital interaction involves a charge transfer from a lone pair (LP) at the interacting F atom of the anion (or HF) to the metal center’s antibonding σ*­(M–C)-orbital. Figure 7 illustrates this LP­(F) → σ*­(M–C) charge transfer, which is observed across all four M–C bonds, though only one is depicted for simplicity. The strength of this donor–acceptor interaction is quantified by the second-order stabilization energy, *E* ^(2)^, which is indicated in Figure 7. For the Group 10 divalent cations, the trend in *E* ^(2)^ values is Ni > Pd > Pt, with values of 11.0 kcal/mol for Ni, 4.3 kcal/mol for Pd, and 2.8 kcal/mol for Pt. This order is consistent with the decreasing electrophilicity observed in the MEP analysis, the weakening of the total binding energy, and the increasing M···F distances observed experimentally. The relatively large *E* ^(2)^ value for the Ni complex confirms it as the strongest π-hole donor among the divalent series in terms of orbital overlap. For the Au^III^ trication, the neutral HF···Au model (Figure 6d) provides an *E* ^(2)^ value of 6.7 kcal/mol. In line with the QTAIM and interaction energy analyses, the charge transfer stabilization is larger for the neutral HF···Au contact (4.0 kcal/mol) compared to the [SbF_6_]^−^···Au anionic contact (2.7 kcal/mol), which further suggest the stronger regium–π interaction for HF. It is noteworthy that the Ni^II^ complex exhibits the highest *E* ^(2)^ value (11.0 kcal/mol) overall, surpassing that of the more electrophilic Au^III^ complex. This is most likely due to the better directionality and more ideal alignment of the Ni···F contact (at 76° to the complex plane), which enables a superior overlap between the F donor lone pair and the metal acceptor σ* orbital. The NBO results thus provide a comprehensive orbital-level rationale for the observed geometric and energetic trends.

**Figure 7:**
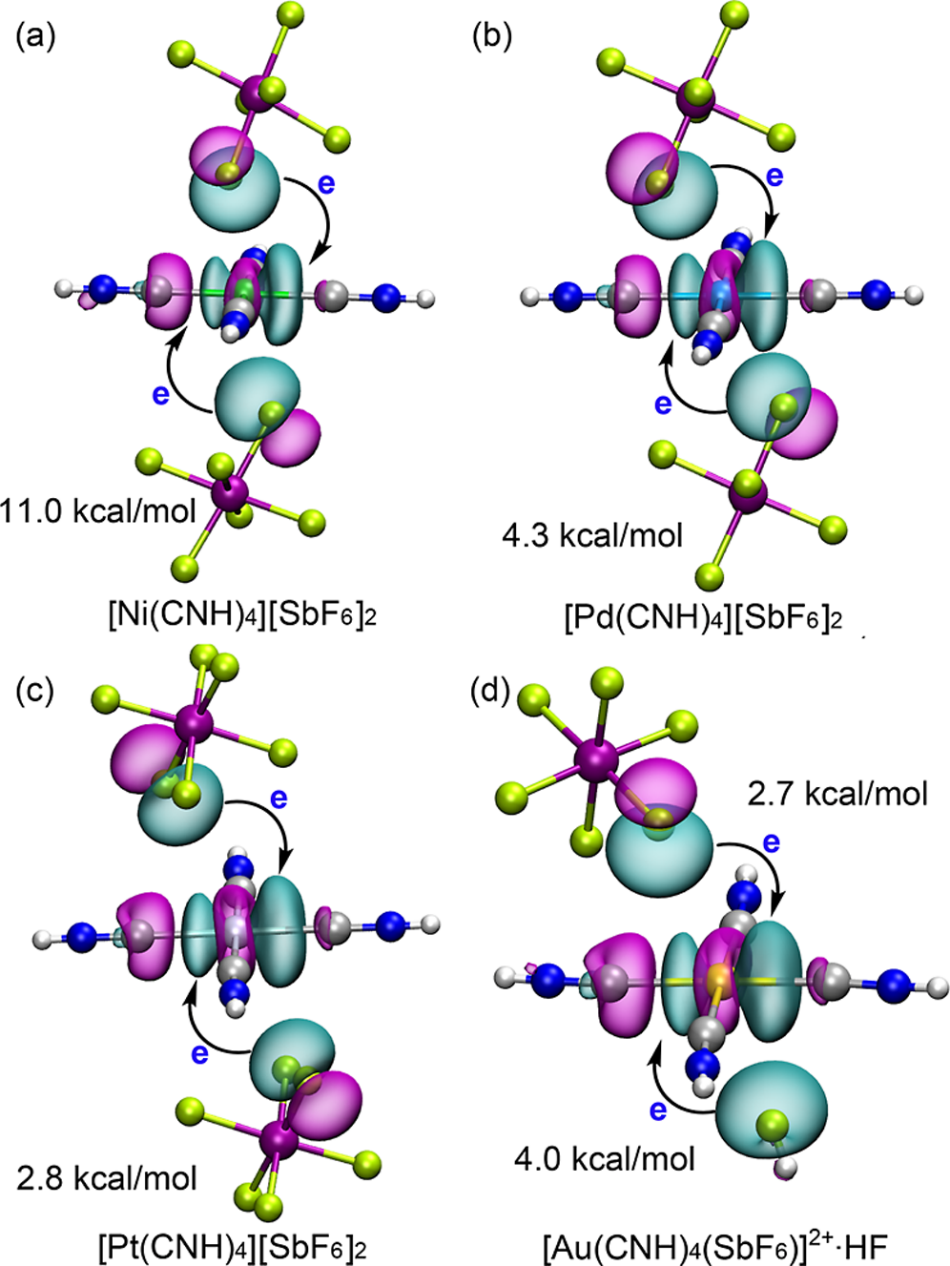
Figure illustrates the LP­(F) → σ*­(M–C) charge transfer orbital interaction, though only one σ* orbital is represented for simplicity. The second-order stabilization energy (*E* ^(2)^ in kcal/mol) is indicated for (a) [Ni­(CNH)_4_]­[SbF_6_]_2_, (b) [Pd­(CNH)_4_]­[SbF_6_]_2_, (c) [Pt­(CNH)_4_]­[SbF_6_]_2_, and (d) the Au^III^ model [Au­(CNH)_4_(SbF_6_)]^2+^·HF.

## Conclusions

We have successfully demonstrated the synthesis and structural characterization of a series of highly charged, homoleptic hydrogen isocyanide complexes, [M­(CNH)_4_]^2+^ (M = Ni, Pd, Pt) and [Au­(CNH)_4_]^3+^, via the 4-fold protonation of their corresponding tetracyanometalate precursors using the superacidic system HF/SbF_5_. The crystal structures unequivocally confirm both the strong H···F hydrogen-bonding networks and the presence of direct, unusually short M···F contacts, establishing these as regium/π-hole interactions. Crucially, the short metal–fluorine distances, especially for the Ni^II^ and Au^III^ complexes, represent a distinct change in the coordination environment of the metal centers. The subsequent DFT investigation provided quantitative and electronic evidence to rationalize the observed structural features. The MEP analysis confirmed the electronic transformation of the typically nucleophilic d^8^ square-planar cores into strong electrophilic sites, demonstrating a “supramolecular Umpolung” where the metal center is switched from an electron donor to a π-hole electron acceptor. The MEP values showed a clear trend of decreasing electrophilicity among the divalent cations from Ni to Pt, which perfectly correlates with the increase in the experimental M···F distances and the corresponding weakening of the ion-pair binding energies. The QTAIM analysis topologically confirmed the presence of M···F bond critical points in all assemblies and, through the ρ­(*r*) values, reinforced the electronic ranking Ni > Pd ≈ Pt for the π-hole strength. Furthermore, the study of the neutral HF···Au model using both QTAIM and energetic analysis disclosed that the intrinsic strength of the regium bond is significantly greater when the long-range Coulombic forces of the [SbF_6_]^−^ counteranion are removed, providing the true estimate of the π-hole’s attractive force. Finally, the NBO analysis quantified the orbital charge transfer from the F lone pair to the metal-centered σ*­(M–C) antibonding orbital. The calculated *E* ^(2)^ values revealed that the Ni^II^ complex exhibits the strongest orbital interaction overall, likely due to a superior orbital overlap geometry, solidifying its position as the most effective π-hole donor among the group 10 divalent metals. This work not only introduces a new class of highly charged, homoleptic metal isocyanide complexes but also provides comprehensive experimental and theoretical evidence for tuning and utilizing the π-hole on a square-planar metal center for supramolecular assembly.

## Experimental Details

The reactions were performed in PFA (tetrafluoroethene-perfluoroalkoxyvinyl-copolymer) tubes with the help of a stainless-steel vacuum line. Caution! SbF_5_ and HF are highly corrosive and toxic compounds with devastating effects on human tissue. They should be handled in appropriate equipment by trained personnel. SbF_5_ was purchased from Sigma-Aldrich and purified via trap-to-trap distillation. Anhydrous HF and SO_2_ are toxic gases. They were stored in stainless steel cylinders. Anhydrous HF was distilled from K_2_NiF_6_ and dried using elemental F_2_ (extreme danger: toxic, corrosive, oxidizing) before use. SO_2_ was stored over CaH_2_. Room temperature (rt) refers to 25 °C. K_2_[Pt­(CN)_4_] and K_2_[Pd­(CN)_4_] were purchased by Sigma-Aldrich and K­[Au­(CN)_4_][Bibr ref48] and K_2_[Ni­(CN)_4_][Bibr ref49] were prepared according to the literature. During all reactions of the polycyanometalates with the superacids, a portable HCN detector was carried to raise the alarm in the event of the possible development of toxic HCN.

### Infrared (IR) Spectroscopy

IR spectra were measured on a FT (Fourier transformation) Nicolet. The sample was directly measured by ATR (attenuated total reflection) technique. Characteristic absorptions are given in wavenumbers ṽ [cm^–1^] and intensities are stated as vs (very strong), s (strong), m (medium) and w (weak). The figures were generated using Origin.^50^

### Raman Spectroscopy

Raman spectra were recorded on a Bruker MultiRAM II equipped with a low-temperature Ge detector (1064 nm). Characteristic absorptions are given in wavenumbers ṽ [cm^–1^] and intensities are stated as vs (very strong), s (strong), m (medium) and w (weak). The figures were generated using Origin.^50^

### Single-Crystal X-ray Diffraction (XRD)

X-ray data were collected on a BRUKER D8 Venture system. Data were collected at 100(2) or 150(2) K using graphite monochromated Mo Kα radiation (λ_α_ = 0.71073 Å). The strategy for the data collection was evaluated by using the Smart software. The data were collected by the standard “ψ–ω scan techniques” and were scaled and reduced using Saint + software. The structure was refined and solved using Olex2.^51^ The structure was solved with the XT^52^ structure solution program using Intrinsic Phasing and refined with the XL refinement package^53,54^ using Least Squares minimization. Bond length and angles were measured with Diamond Crystal and Molecular Structure Visualization Version 4.6.2.^55^ Drawings were generated with POV-Ray.^56^

### Tetrakis­(hydrogenisocyanide)­gold­(III) Hexafluoroantimonate [Au­(CNH)_4_]­[SbF_6_]_3_

Antimony pentafluoride (200 mg, 0.922 mmol, 15 equiv) was filled into an 8 mm PFA tube equipped with a stainless-steel valve. Anhydrous HF (0.8 mL) was condensed in at −196 °C. The mixture was warmed to room temperature and shaken, after which it was cooled to −196 °C again. Potassium tetracyanoaurate­(III) (20 mg, 0.059 mmol, 1 equiv) was added to the frozen mixture and warmed to room temperature. A colorless suspension was received. Sulfur dioxide (0.2 mL) was condensed at −196 °C to the mixture, which resulted in a colorless solution at room temperature. The mixture was slowly cooled to −78 °C in a freezer. Moisture-sensitive off-white crystals of [Au­(CNH)_4_]­[SbF_6_]_3_·2HF were received.

**FT-IR** (ATR) ṽ [cm^–1^] = 3148 (m), 2212 (w), 1617 (w), 678 (vs), 650 (vs), 486 (s).

**Raman** ṽ [cm^–1^] = due to fluorescence, no analyzable signal could be detected.

### Tetrakis­(hydrogenisocyanide)­nickel­(II) Hexafluoroantimonate [Ni­(CNH)_4_]­[SbF_6_]_2_

The reaction was performed analogously to the one reported for [Au­(CNH)_4_]­[SbF_6_]_3_.

**FT-IR** (ATR) ṽ [cm^–1^] = 3225 (m), 2992 (m), 2181 (vw), 1662 (w), 830 (w), 686 (vs), 652 (vs), 484 (s).

**Raman** ṽ [cm^–1^] = 2195 (s), 669 (s), 660 (s), 292 (m), 138 (w).

### Tetrakis­(hydrogenisocyanide)­palladium­(II) Hexafluoroantimonate [Pd­(CNH)_4_]­[SbF_6_]_2_

The reaction was performed analogously to the one reported for [Au­(CNH)_4_]­(SbF_6_)_3_.

**FT-IR** (ATR) ṽ [cm^–1^] = 3279 (m), 3179 (m), 2190 (vw), 1616 (w), 679 (vs), 651 (vs), 484 (s).

**Raman** ṽ [cm^–1^] = 2213 (s), 2199 (s), 686 (m), 659 (vs), 297 (m), 230 (m), 131 (m), 92 (m).

### Tetrakis­(hydrogenisocyanide)­platinum­(II) Hexafluoroantimonate [Pt­(CNH)_4_]­[SbF_6_]_2_

The reaction was performed analogously to the one reported for [Au­(CNH)_4_]­[SbF_6_]_3_.

**FT-IR** (ATR) ṽ [cm^–1^] = 3307 (m), 2190 (w), 1612 (w), 683 (vs), 483 (s), 457 (m).

**Raman** ṽ [cm^–1^] = 2221 (vs), 2205 (vs), 658 (vs), 296 (m), 242 (w), 132 (m), 90 (m).

## Computational Methods

All density functional theory (DFT) calculations were performed using the Turbomole 7.7 program package.^57^ The calculations were carried out on simplified cluster models derived directly from the experimental geometries determined by scXRD, which allowed for the study of the noncovalent interactions as they exist in the solid state. The hybrid functional PBE0 ^58^ was selected to include a fraction of exact exchange, and it was combined with the latest version of the empirical dispersion correction, D4,^59^ to accurately model the noncovalent interactions.^58^ The def2-TZVP basis set was employed for all atoms.^60^ Importantly, for the heavier elements (Pd, Pt, Au, and Sb), this basis set utilizes Effective Core Potentials (ECPs), which implicitly account for scalar relativistic effects, a critical necessity for accurately describing the electronic structure of 4d and 5d transition metals.^61^

The nature and strength of the observed noncovalent interactions were further analyzed using electronic structure methods. Molecular electrostatic potential (MEP) surfaces were computed on the 0.001 au isodensity surface to visualize the π-hole on the metal centers. The quantum theory of atoms in molecules (QTAIM)^62^ analysis was performed using the MultiWFN 3.8 ^63^ program to identify bond critical points (BCPs) and bond paths, topologically confirming the attractive interactions. Finally, the electronic charge transfer and donor–acceptor interactions were quantified using Natural Bond Orbital (NBO) analysis,^64^ as implemented in the NBO7 program, to obtain the second-order perturbation energies (*E* ^(2)^).

## Supplementary Material



## Notes

Spectroscopic and crystallographic data. Crystallographic data have been deposited with the Cambridge Crystallographic Data Centre (CCDC) with identifiers 2500044–2500047. Copies of the data can be obtained free of charge on application to the CCDC (PDF)

## References

[1] Alexandrov E. V., Virovets A. V., Blatov V. A., Peresypkina E. V. (2015). Topological
Motifs in Cyanometallates: From Building Units to Three-Periodic Frameworks. Chem. Rev..

[2] Lu Y., Wang L., Cheng J., Goodenough J. B. (2012). Prussian
blue: a new framework of electrode materials for sodium batteries. Chem. Commun..

[3] Avila Y., Acevedo-Peña P., Reguera L., Reguera E. (2022). Recent progress in
transition metal hexacyanometallates: From structure to properties
and functionality. Coord. Chem. Rev..

[4] Xie Y., Lin R.-B., Chen B. (2022). Old Materials for New Functions:
Recent Progress on Metal Cyanide Based Porous Materials. Adv. Sci..

[5] Entley W. R., Treadway C. R., Girolami G. S. (1995). Molecular
Magnets Constructed from
Cyanometalate Building Blocks. Mol. Cryst. Liq.
Cryst..

[6] Atanasov M., Comba P., Hausberg S., Martin B. (2009). Cyanometalate-bridged
oligonuclear transition metal complexes—Possibilities for a
rational design of SMMs. Coord. Chem. Rev..

[7] Derossi S., Brammer L., Hunter C. A., Ward M. D. (2009). Halogen Bonded Supramolecular
Assemblies of [Ru­(bipy)­(CN)_4_]^2‑^ Anions
and N-Methyl-Halopyridinium Cations in the Solid State and in Solution. Inorg. Chem..

[8] Ormond-Prout J. E., Smart P., Brammer L. (2012). Cyanometallates
as Halogen Bond Acceptors. Cryst. Growth Des..

[9] Sellin M., Rupf S. M., Zhang Y., Malischewski M. (2020). Bi- and Trifurcated
Halogen Bonding M–C≡N···I in 1D, 2D,
and 3D Supramolecular Network Structures of Co-Crystallized Diiodoacetylene
C_2_I_2_ and Tetracyanonickelate [Ni­(CN)_4_]^2–^. Cryst. Growth Des..

[10] Sellin M., Rupf S. M., Malischewski M. (2021). Cubic Three-Dimensional
Networks
of the Cyanometalate [Fe­(CN)_6_]^3–^ with
the Ditopic Halogen Bond Donor Diiodoacetylene C_2_I_2_. Cryst. Growth Des..

[11] Streit T.-N., Gomila R. M., Sievers R., Frontera A., Malischewski M. (2024). CF_3_-substituted sulfonium cations as efficient chalcogen bond donors
towards cyanometalates. CrystEngComm.

[12] Streit T.-N., Langwald J., Gomila R. M., Frontera A., Malischewski M. (2024). Structural
diversity of supramolecular networks formed between polycyanometalates
and sulfur-based chalcogen bond donors. CrystEngComm.

[13] Cvrtila I., Stilinović V. (2017). New Tricks by Old Anions: Hydrogen Bonded Hexacyanoferrous
Anionic Networks. Cryst. Growth Des..

[14] Tanaka R., Okazawa A., Kojima N., Matsushita N. (2018). Ionic Crystal
Containing Protons (H+) as Counter Cations: Preparation and Crystal
Structure of a Salt of 4,4′-Bipiperidine-1,1′-diium
and Hexacyanidoferrate­(II). Chem. Lett..

[15] Gorelsky S. I., Ilyukhin A. B., Kholin P. V., Kotov V. Y., Lokshin B. V., Sapoletova N. V. (2007). Dihydrohexacyanoferrates of N-heterocyclic cations. Inorg. Chim. Acta.

[16] Xydias P., Lymperopoulou S., Dokorou V., Manos M., Plakatouras J. C. (2019). Supramolecular
networks derived from hexacyanoferrates and nitrogen heterocyclic
cations. Polyhedron.

[17] Sellin M., Marvaud V., Malischewski M. (2020). Isolation
and Structural Characterization
of Eightfold Protonated Octacyanometalates [M­(CNH)_8_]^4+^ (M = Mo^IV^,W^IV^) from Superacids. Angew. Chem., Int. Ed..

[18] Jakupec N., Fotović L., Stilinović V. (2020). The effect of halogen bonding on
protonated hexacyanoferrate networks in hexacyanoferrates of halogenopyridines. CrystEngComm.

[19] Carvalho M. F. N. N., Galvão A. M., Pombeiro A. J. L. (2000). Proton addition
and hydrogen-bond formation in reactions of the dicyano-complex [NBu_4_]­[trans-Re­(CN)_2_(dppe)_2_] with protic
reagents. J. Chem. Soc., Dalton Trans..

[20] Pombeiro A. J. L. (2001). Coordination
chemistry of CNH, the simplest isocyanide. Inorg.
Chem. Commun..

[21] Rieger D., Hahn F. E., Fehlhammer W. P. (1990). The supercomplex
nature of Buff’s
″ferrocenäthyl″. First example of a homoleptic
hydrogen isocyanide (CNH) metal complex. J.
Chem. Soc. Chem. Commun..

[22] Scheiner S. (2025). The Next Frontier
in the Study of Noncovalent Bonding: Transition Metals. Molecules.

[23] Alkorta I., Elguero J., Frontera A. (2020). Not Only Hydrogen
Bonds: Other Noncovalent
Interactions. Crystals.

[24] Frontera A., Bauzá A. (2018). Regium−π bonds: An Unexplored Link between
Noble Metal Nanoparticles and Aromatic Surfaces. Chem. - Eur. J..

[25] Halldin
Stenlid J., Johansson A. J., Brinck T. (2018). σ-Holes and σ-lumps
direct the Lewis basic and acidic interactions of noble metal nanoparticles:
introducing regium bonds. Phys. Chem. Chem.
Phys..

[26] Zierkiewicz W., Michalczyk M., Scheiner S. (2018). Regium bonds between Mn clusters
(M = Cu, Ag, Au and n = 2–6) and nucleophiles NH_3_ and HCN. Phys. Chem. Chem. Phys..

[27] Sánchez-Sanz G., Trujillo C., Alkorta I., Elguero J. (2019). Understanding Regium
Bonds and their Competition with Hydrogen Bonds in Au_2_:HX
Complexes. ChemPhysChem.

[28] Scheiner S. (2025). Semicoordinate
and halogen bonding to group 10 and group 8 metals. Phys. Chem. Chem. Phys..

[29] Amonov A., Scheiner S. (2025). Semicoordinate versus
σ-Hole Bonding of Group
10 Metal Atoms in a Square Planar Motif. Inorg.
Chem..

[30] Terrón A., Buils J., Mooibroek T. J., Barceló-Oliver M., García-Raso A., Fiol J. J., Frontera A. (2020). Synthesis, X-ray characterization
and regium bonding interactions of a trichlorido­(1-hexylcytosine)­gold­(iii)
complex. Chem. Commun..

[31] Yan J., Zeng Y., Meng L., Li X., Zhang X. (2023). Gold­(iii)
derivatives as the noncovalent interaction donors: theoretical study
of the π-hole regium bonds. Phys. Chem.
Chem. Phys..

[32] Pizzi A., Calabrese M., Daolio A., Ursini M., Frontera A., Resnati G. (2022). Expanding
the toolbox of the coinage bond: adducts
involving new gold­(iii) derivatives and bioactive molecules. CrystEngComm.

[33] Andreo L., Gomila R. M., Priola E., Giordana A., Pantaleone S., Diana E., Mahmoudi G., Frontera A. (2022). Anion···Anion
[AuI_4_]^−^···[AuI_2_]^−^ Complex Trapped in the Solid State by Tetramethylammonium
Cations. Cryst. Growth Des..

[34] Giordana A., Priola E., Mahmoudi G., Doustkhah E., Gomila R. M., Zangrando E., Diana E., Operti L., Frontera A. (2025). Exploring coinage bonding interactions in [Au­(CN)_4_]^−^ assemblies with silver and zinc complexes:
a structural and theoretical study. Phys. Chem.
Chem. Phys..

[35] de
las Nieves Piña M., Mooibroek T. J., Frontera A., Bauzá A. (2022). Importance of Cu and Ag regium−π
bonds in supramolecular chemistry and biology: a combined crystallographic
and ab initio study. Phys. Chem. Chem. Phys..

[36] Burguera S., Frontera A., Bauza A. (2023). Regium−π Bonds Involving
Nucleobases: Theoretical Study and Biological Implications. Inorg. Chem..

[37] Piña M. d. l.
N., Frontera A., Bauzá A. (2020). Regium−π Bonds Are Involved
in Protein–Gold Binding. J. Phys. Chem.
Lett..

[38] Buils J., Terrón A., Barceló-Oliver M., Fiol J. J., García-Raso A., Gomila R. M., Frontera A. (2025). Synthesis, X-ray characterization,
and DFT calculations of gold–nucleobase complexes: on the importance
of regium bonds and anion−π interactions. CrystEngComm.

[39] Barwise L., Moon L. J., Dhakal B., Hogan C. F., White K. F., Dutton J. L. (2024). An extremely electron
poor Au­(iii) trication bearing
acetonitrile ligands. Chem. Commun..

[40] Corbo R., Pell T. P., Stringer B. D., Hogan C. F., Wilson D. J. D., Barnard P. J., Dutton J. L. (2014). Facile Formation of Homoleptic Au­(III)
Trications via Simultaneous Oxidation and Ligand Delivery from [PhI­(pyridine)_2_]^2+^. J. Am. Chem. Soc..

[41] Albayer M., Dutton J. L. (2019). Synthesis of cationic gold­(III) complexes using iodine­(III). J. Coord. Chem..

[42] Ptasiewicz-Bak H., Olovsson I., McIntyre G. J. (1998). Structure, Charge and Spin Density
in Na_2_Ni­(CN)_4_·3H_2_O at 295 and
30 K. Acta Crystallogr..

[43] Mühle C., Nuss J., Dinnebier R. E., Jansen M. (2004). Über Kaliumtetracyanoplatinat­(II),
Kaliumtetracyanopalladat­(II) und deren Monohydrate. Z. Anorg. Allg. Chem..

[44] Bertinotti C., Bertinotti A. (1970). Structure cristalline de l’auricyanure de potassium
monohydraté par la diffraction des neutrons. Acta Crystallogr..

[45] Li Y., Liu L., Jia D., Guo J., Sheng R. (2011). Synthesis Crystal Structures
and Fluorescent Properties of Two Bimetallic Coordination Polymers. J. Inorg. Organomet. Polym. Mater..

[46] Batsanov S. S. (2001). Van der
Waals Radii of Elements. Inorg. Mater..

[47] Hu S.-Z., Zhou Z.-H., Xie Z.-X., Robertson B. E. (2014). A comparative
study of crystallographic van der Waals radii. Z. Kristallogr..

[48] Bernhardt E., Finze M., Willner H. (2004). Synthesis
and NMR spectroscopic investigation
of salts containing the novel [Au­(CF_3_)_n_X_4–n_]– (n = 4–1, X = F, CN, Cl) anions. J. Fluorine Chem..

[49] Kürkcüoglu G. S., Karaagac D., Yesilel O. Z., Tas M. (2012). Synthesis, Spectroscopic
and Structural Properties of Heteropolynuclear Cyano-Bridged Complexes. J. Inorg. Organomet. Polym. Mater..

[50] OriginPro, Version; OriginLab Corporation: Northhampton, MA, USA, 2024.

[51] Dolomanov O. V., Bourhis L. J., Gildea R. J., Howard J. A. K., Puschmann H. (2009). OLEX2: a complete
structure solution, refinement and analysis program. J. Appl. Crystallogr..

[52] Sheldrick G. M. (2015). SHELXT
– Integrated space-group and crystal-structure determination. Acta Crystallogr..

[53] Sheldrick G. M. (2015). Crystal
structure refinement with SHELXL. Acta Crystallogr..

[54] SHELXL Version 2014/7; Program for Crystal Structure solution and Refinement: Göttingen, Germany, 2014.

[55] Crystal Impact - Dr. H. Putz and Dr. K. Brandenburg GbR . Diamond - Crystal and Molecular Structure Visualization: Bonn, Germany.

[56] Povray; Persistence of Vision Pty. Ltd . Persistence of Vision Raytracer; Persistence of Vision Pty. Ltd, 2004. http://www.povray.org/download/.

[57] Ahlrichs R., Bär M., Häser M., Horn H., Kölmel C. (1989). Electronic
structure calculations on workstation computers: The program system
turbomole. Chem. Phys. Lett..

[58] Adamo C., Barone V. (1999). Toward reliable density functional
methods without
adjustable parameters: The PBE0 model. J. Chem.
Phys..

[59] Caldeweyher E., Bannwarth C., Grimme S. (2017). Extension of the D3 dispersion coefficient
model. J. Chem. Phys..

[60] Weigend F., Ahlrichs R. (2005). Balanced basis sets
of split valence, triple zeta valence
and quadruple zeta valence quality for H to Rn: Design and assessment
of accuracy. Phys. Chem. Chem. Phys..

[61] Andrae D., Häußermann U., Dolg M., Stoll H., Preuß H. (1990). Energy-adjusted ab initio pseudopotentials for the
second and third row transition elements. Theor.
Chim. Acta.

[62] Bader R. F. W. (1991). A quantum
theory of molecular structure and its applications. Chem. Rev..

[63] Lu T., Chen F. (2012). Multiwfn A Multifunctional
Wavefunction Analyzer. J. Comput. Chem..

[64] Glendening E. D., Landis C. R., Weinhold F. (2019). NBO 7.0: New vistas in localized
and delocalized chemical bonding theory. J.
Comput. Chem..

