# Supplementary material for: 4‑Fold Protonation of Tetracyanometalates in Superacids: Hydrogen and π‑Hole Bonding in the Solid State
Source: Inorg Chem. 2025 Dec 29;65(1):892–901. doi: 10.1021/acs.inorgchem.5c05224 (PMC12801322; doi:10.1021/acs.inorgchem.5c05224)
Supplement: Supplementary file 1 [file ic5c05224_si_001.pdf]

Supporting Information

4-Fold Protonation of Tetracyanometalates in Superacids: Hydrogen and π-Hole Bonding in the Solid State

Tim-Niclas Streit,[a] Malte Sellin,[b] Susanne M. Rupf,[a] Rosa M. Gomila,[c] Antonio Frontera,[c*] Moritz Malischewski[a*]

[a] Freie Universität Berlin
Institut für Chemie und Biochemie
Fabeckstraße 34–36
14195 Berlin, Germany
moritz.malischewski@fu-berlin.de

[b] University of Basel
Department of Chemistry
St. Johanns-Ring 19
4056 Basel, Switzerland

[c]Universitat de les Illes Balears
Departament de Quimica
Crta. de Valldemossa km 7.5
07122 Palma de Mallorca (Baleares), Spain
toni.frontera@uib.es

Table of Contents

1. Analytical Data S2
1.1 IR Spectroscopy S2
1.2 Raman Spectroscopy S4
2. Crystallographic Data S6

1. Analytical Data

1.1 IR Spectroscopy

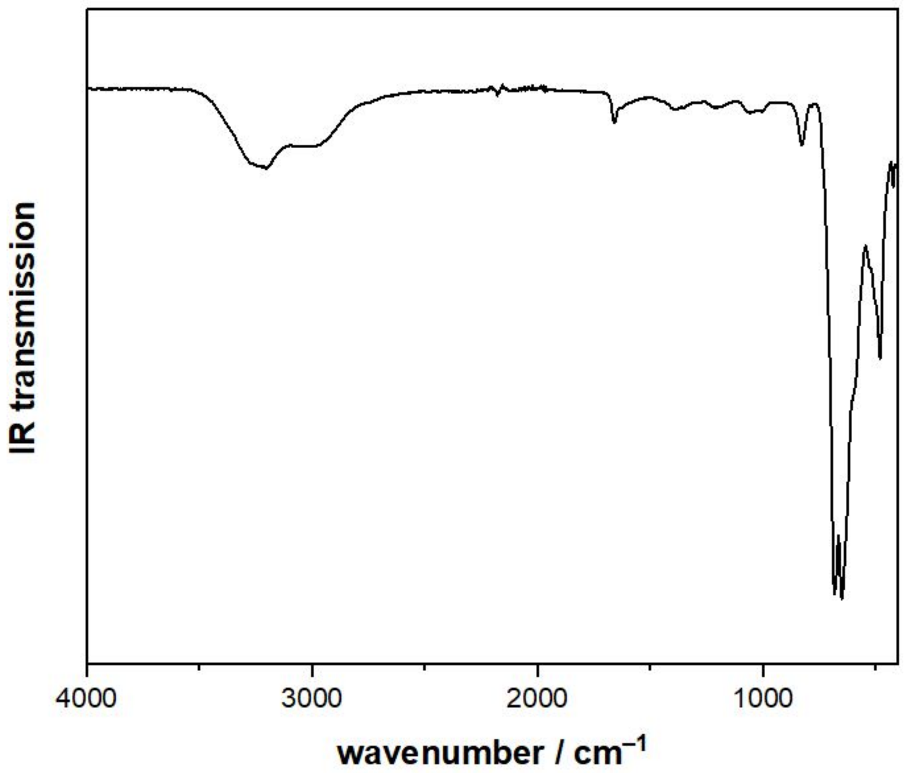

Figure S1. Experimental IR (ATR, rt) spectrum of $[Ni(CNH)_4][SbF_6]_2$.

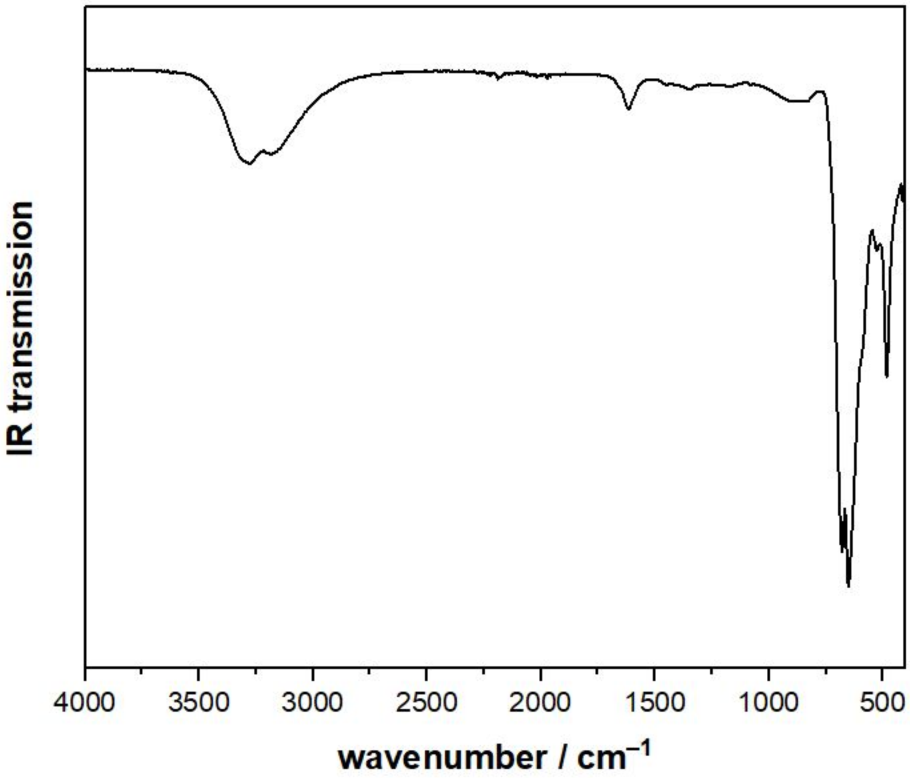

Figure S2. Experimental IR (ATR, rt) spectrum of $[Pd(CNH)_4][SbF_6]_2$.

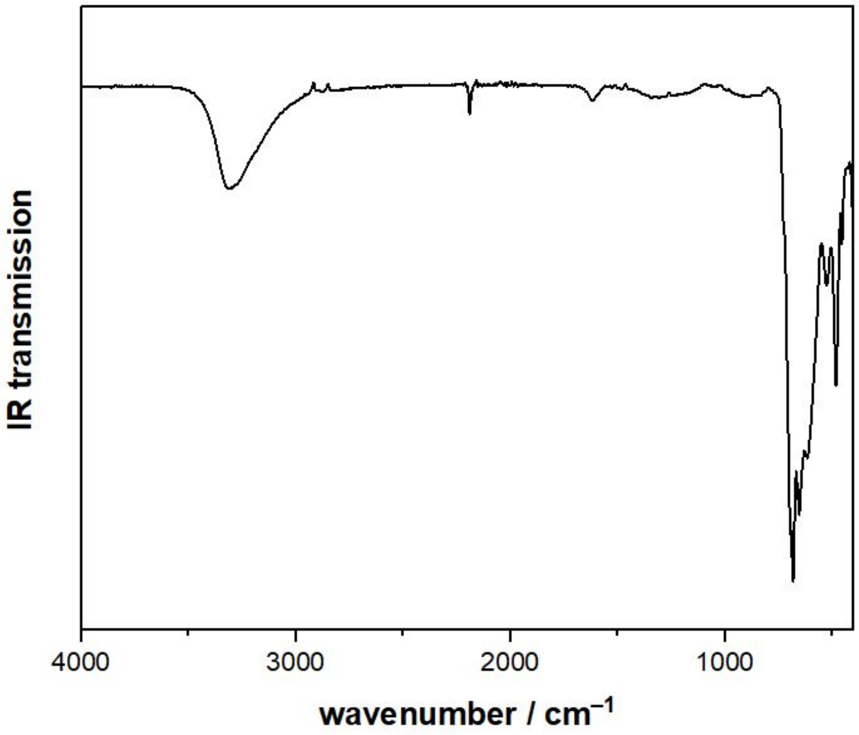

Figure S3. Experimental IR (ATR, rt) spectrum of $[Pt(CNH)_4][SbF_6]_2$.

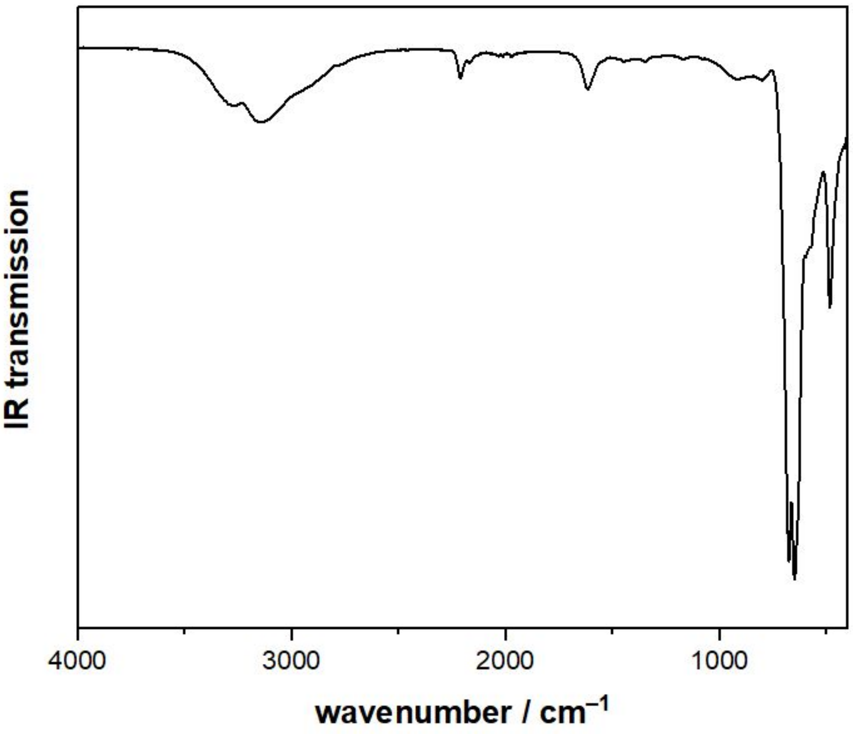

Figure S4. Experimental IR (ATR, rt) spectrum of $[Au(CNH)_4][SbF_6]_3$.

1.2 Raman Spectroscopy

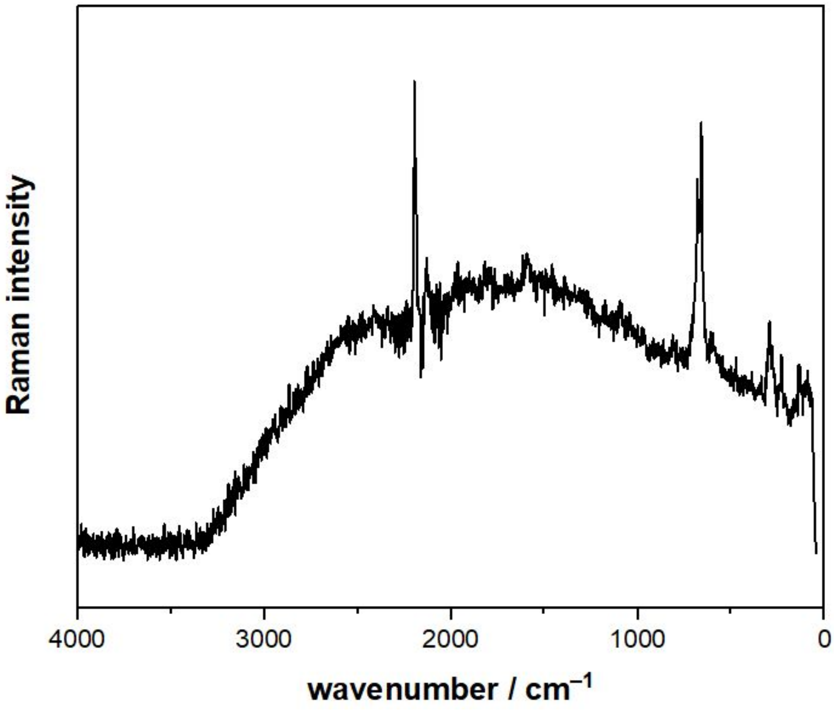

Figure S5. Experimental Raman (rt) spectrum of $[Ni(CNH)_4][SbF_6]_2$.

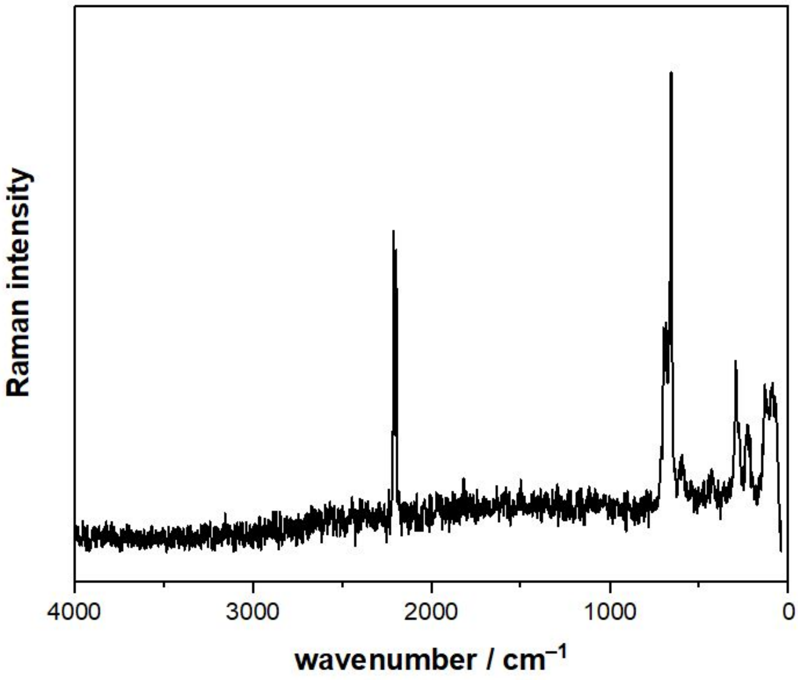

Figure S6. Experimental Raman (rt) spectrum of $[Pd(CNH)_4][SbF_6]_2$.

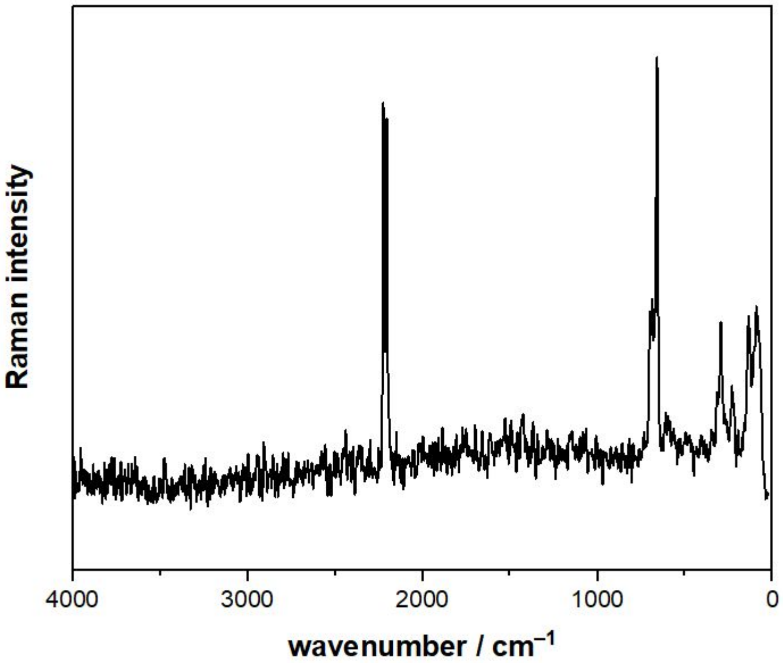

Figure S7. Experimental Raman (rt) spectrum of $[Pt(CNH)_4][SbF_6]_2$.

2. Crystallographic Data

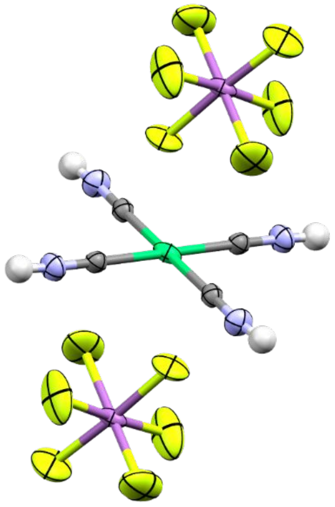

Figure S8. Molecular structure in solid state of $[Ni(CNH)_4][SbF_6]_2$. Ellipsoids are shown at 50% probability. Ni green, Sb purple, F yellow, C grey, N pastel blue.

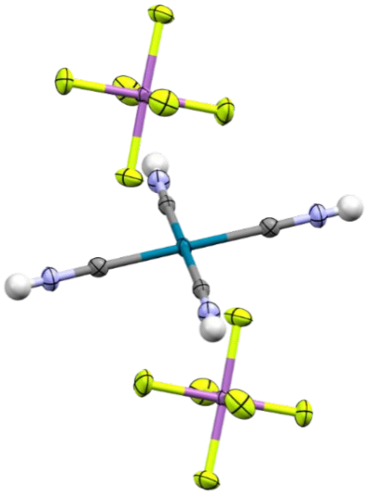

Figure S9. Molecular structure in solid state of $[Pd(CNH)_4][SbF_6]_2$. Ellipsoids are shown at 50% probability. Pd dark green, Sb purple, F yellow, C grey, N pastel blue.

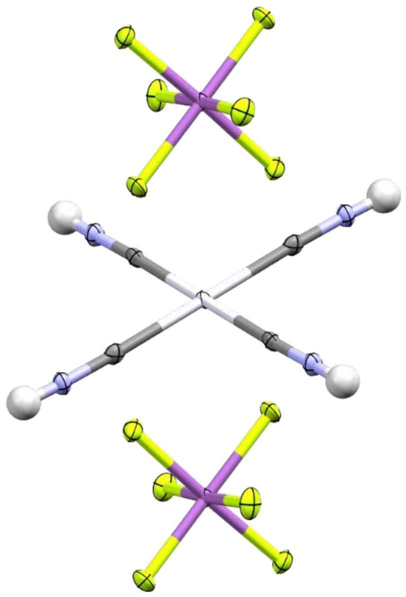

Figure S10. Molecular structure in solid state of $[Pt(CNH)_4][SbF_6]_2$.Ellipsoids are shown at 50% probability. Pt white, Sb purple, F yellow, C grey, N pastel blue.

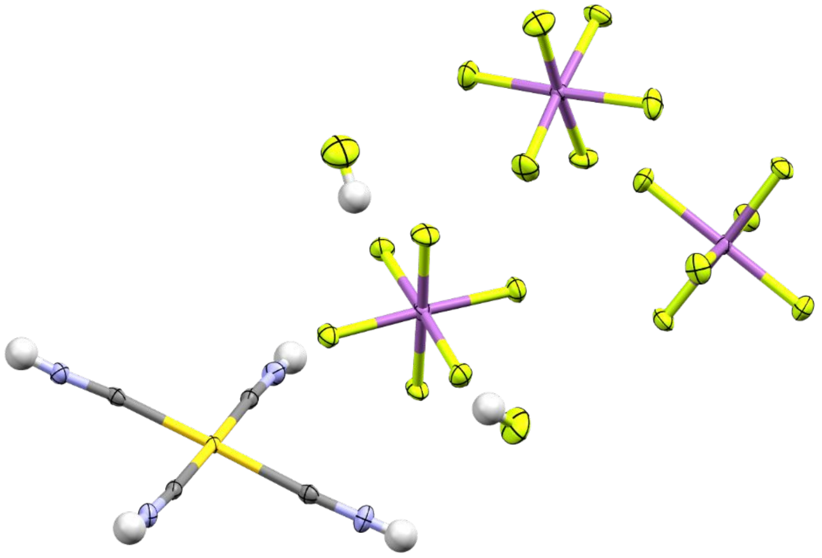

Figure S11. Molecular structure in solid state of $[Au(CNH)_4][SbF_6]_3{\cdot}2HF$. Ellipsoids are shown at 50% probability. Au yellow, Sb purple, F yellow, C grey, N pastel blue.

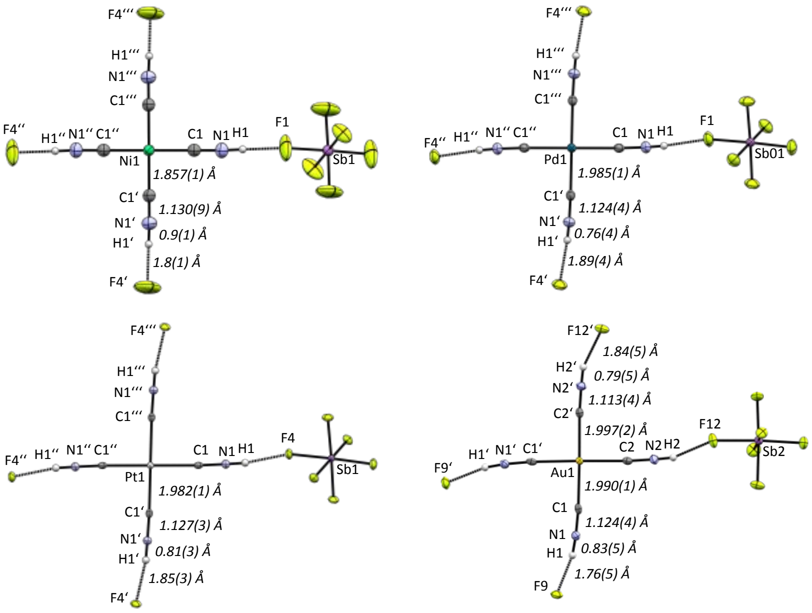

Figure S12. Selected bond lengths in the solid-state structures of the protonated cyanometalates.

Table S1. Crystallographic data of $[Ni(CNH)_4][SbF_6]_2$ (left) and $[Pd(CNH)_4]$ $[SbF_6]_2$ (right).

CCDC Identifier	2500044	2500045
Empirical formula	$C_4H_4F_{12}N_4NiSb_2$	$C_4H_4F_{12}N_4PdSb_2$
Formula weight	638.32	686.01
Temperature/K	150.00	150.00
Crystal system	monoclinic	monoclinic
Space group	C2/m	C2/m
a/Å	12.9457(12)	12.0949(7)
b/Å	10.3518(10)	10.0935(6)
c/Å	6.3151(5)	6.9904(4)
α/°	90	90
β/°	115.402(3)	116.9850(10)
γ/°	90	90
Volume/Å^3	764.48(12)	760.47(8)
Z	2	2
ρ_{calc}g/cm^3	2.773	2.996
μ/mm^{-1}	4.854	4.825
F(000)	588.0	624.0
Crystal size/mm^3	0.557 × 0.1 × 0.074	0.361 × 0.345 × 0.162
Radiation	MoKα (λ = 0.71073)	MoKα (λ = 0.71073)
2Θ range for data collection/°	5.256 to 50.702	5.53 to 50.706
Index ranges	-15 ≤ h ≤ 15, -12 ≤ k ≤ 12, -6 ≤ l ≤ 7	-14 ≤ h ≤ 14, -12 ≤ k ≤ 12, -8 ≤ l ≤ 8
Reflections collected	5416	10038
Independent reflections	744 [R_{int} = 0.0484, R_{sigma} = 0.0255]	741 [R_{int} = 0.0391, R_{sigma} = 0.0150]
Data/restraints/parameters	744/0/63	741/0/63
Goodness-of-fit on F^2	1.187	1.232
Final R indexes [I>=2σ (I)]	R_1 = 0.0313, wR_2 = 0.0678	R_1 = 0.0140, wR_2 = 0.0370

Table S2. Crystallographic data of $[Pt(CNH)_4](SbF_6)_2$ (left) and $[Au(CNH)_4][SbF_6]_3 \cdot 2HF$ (right).

CCDC Identifier	2500046	2500047
Empirical formula	$C_4H_4F_{12}N_4PtSb_2$	$C_4H_6AuF_{20}N_4Sb_3$
Formula weight	774.70	1052.333
Temperature/K	100.00	100.00
Crystal system	monoclinic	Orthorhombic
Space group	I2/m	Pnma
a/Å	7.1149(6)	15.906(2)
b/Å	9.9659(8)	9.1567(13)
c/Å	10.6807(11)	14.523(2)
α/°	90	90
β/°	99.451(3)	90
γ/°	90	90
Volume/Å^3	747.05(12)	2115.2(5)
Z	2	4
ρ_{calc}g/cm^3	3.444	3.305
μ/mm^{-1}	13.061	10.877
F(000)	688.0	1869.2
Crystal size/mm^3	0.17 × 0.15 × 0.15	0.24 × 0.24 × 0.2
Radiation	MoKα (λ = 0.71073)	MoKα (λ = 0.71073)
2Θ range for data collection/°	5.626 to 56.654	3.8 to 53.2
Index ranges	-9 ≤ h ≤ 9, -13 ≤ k ≤ 13, -14 ≤ l ≤ 14	-19 ≤ h ≤ 19, -11 ≤ k ≤ 11, -18 ≤ l ≤ 18
Reflections collected	14220	67987
Independent reflections	994 [R_{int} = 0.0266, R_{sigma} = 0.0104]	2324 [R_{int} = 0.0451, R_{sigma} = 0.0118]
Data/restraints/parameters	994/0/63	2324/2/179
Goodness-of-fit on F^2	1.142	1.053
Final R indexes [I>=2σ (I)]	R_1 = 0.0105, wR_2 = 0.0253	R_1 = 0.0148, wR_2 = 0.0299
Final R indexes [all data]	R_1 = 0.0106, wR_2 = 0.0253	R_1 = 0.0172, wR_2 = 0.0307
